# ADHFE1 is a correlative factor of patient survival in cancer

**DOI:** 10.1515/biol-2021-0065

**Published:** 2021-06-18

**Authors:** Qi Chen, Qiyan Wu, Yaojun Peng

**Affiliations:** Department of Traditional Chinese Medicine, The First Medical Centre, Chinese PLA General Hospital, Beijing, China; Cancer Center Key Lab, The First Medical Centre, Chinese PLA General Hospital, Beijing, China; Department of Emergency, The First Medical Centre, Chinese PLA General Hospital, #28 Fuxing Road, Beijing 100853, China

**Keywords:** ADHFE1, cancer biomarker, bioinformatics analysis, DNA methylation

## Abstract

Alcohol dehydrogenase iron containing 1 (ADHFE1) encodes a hydroxyacid-oxoacid transhydrogenase participating in multiple biological processes. The role of ADHFE1 in cancer has not been fully uncovered. Herein, we performed data analysis to investigate the expression of ADHFE1 and the underlying regulatory mechanisms, its relationship with cancer patients’ survival, and the relevant pathways in cancer. A range of recognized, web-available databases and bioinformatics tools were used in this *in silico* study. We found that ADHFE1 was frequently downregulated and hypermethylated in various cancer cell lines and tissue samples. High expression of ADHFE1 was positively associated with favorable patient prognosis in breast, colon, and gastric cancers. Pathway analysis revealed its potential role in cancer-related biological processes, including energy metabolism, DNA replication, and cell cycle regulation. AHDFE1 mRNA expression and DNA methylation can potentially be used as diagnostic markers in cancer and might be of great value in predicting the survival of patients with cancer.

## 1 Introduction

Cancer is expected to rank as the leading cause of death and the single most barrier to increasing life expectancy worldwide [1]. The incidence and mortality of cancer are steadily growing due to complex reasons, including aging, population growth, and changes in the prevalence and distribution of the main risk factors of cancer [1]. Despite improvement in cancer diagnosis and treatment, the overall prognosis is still unsatisfactory. Therefore, effective diagnostic, prognostic, and predictive biomarkers are clearly and urgently needed. It is widely acknowledged that gene expression is commonly regulated by genetic and epigenetic mechanisms, and the accumulation of genetic and epigenetic alterations is a crucial event contributing to oncogenesis [2]. Identification of differentially expressed genes between cancer and normal tissues and uncovering the underlying regulatory mechanisms as well as their functional roles in cancer initiation and development can aid in the discovery of novel biomarkers for early diagnosis, survival prediction, and target therapy.

Alcohol dehydrogenase iron containing 1 (ADHFE1) was first cloned and characterized by Deng from the human fetal brain cDNA library [3]. ADHFE1 encodes a hydroxyacid-oxoacid transhydrogenase, which belongs to the group Ⅲ metal-dependent alcohol dehydrogenase family, and it mainly participates in the process of 4-hydroxybutyrate oxidation to succinate semialdehyde [4,5]. On the cellular level, ADHFE1 is localized in mitochondria and exhibits differentiation-dependent expression during *in vitro* brown and white adipogenesis, indicating its role in adipocyte function and energy metabolism [6]. The role of ADHFE1 in cancer is not fully uncovered. In esophageal squamous cell carcinoma, ADHFE1 was proposed as a hypermethylated tumor suppressor gene in a Chinese Han population [7]. It was reported that in colorectal cancer (CRC), ADHFE1 was hypermethylated, and a high expression level of ADHFE1 was positively associated with tumor differentiation, indicating its tumor-suppressing function in CRC [8]. More recently, hypermethylation of ADHFE1 has been revealed to promote the proliferation of CRC cells via modulating cell cycle progression [9]. However, ADHFE1 has been reported to form a mutual regulatory loop with MYC, and ADHFE1 may play an oncogenic role in breast cancer via inducing metabolic reprogramming [10].

In this study, we systematically examined the expression of ADHFE1 and the regulatory mechanisms of ADHFE1 expression in cancer cell lines and tissue samples. Additionally, we evaluated the prognostic value of ADHFE1 in certain cancer types (breast, colon, and gastric cancers) using several cancer datasets. Finally, ADHFE1-associated pathways and biological processes were explored to unveil the potential functions and molecular mechanisms of ADHFE1 in cancer.

## 2 Materials and methods

### 2.1 ADHFE1 expression and methylation in cancer cell lines

Based on the NCI-60 cell line set, CellMiner (https://discover.nci.nih.gov/cellminer) is a web-based pharmacologic and genomic tool to explore transcript and drug patterns in a panel of recognized cancer cell lines [11,12]. Integrated data of whole-exome sequencing, gene and miRNA transcripts, DNA copy number, DNA methylation, and protein levels of the NCI-60 cell lines were included in the database [11,12]. Using CellMiner, we examined the relativity between mRNA expression and DNA methylation of ADHFE1 in the NCI-60 cell lines.

### 2.2 Transcript expression analysis using Oncomine and Gene Expression across Normal and Tumor tissue (GENT)

Based on microarray datasets, Oncomine (http://www.oncomine.org) is a helpful cancer database delivering standardized transcriptome data for cancer researchers [13]. The mRNA expression levels of ADHFE1 in various types of cancer tissues and their normal counterparts were inquired in the Oncomine database with the threshold parameters of |fold-change| >2 and *P*-value <0.05. GENT (http://medical-genome.kribb.re.kr/GENT/) is another online database providing gene expression patterns across a number of cancer and normal tissues [14]. Using the default searching criterion of the GENT database, we validated the differential gene expression pattern observed in the Oncomine database.

### 2.3 Transcript expression analysis using Gene Expression Profiling Interactive Analysis (GEPIA)

Based on The Cancer Genome Atlas (TCGA) and Genotype-Tissue Expression (GTEx) data, GEPIA (http://gepia.cancer-pku.cn/) delivers fast and customizable functionalities, including differential expression analysis, patient survival analysis, profiling plotting, correlation analysis, similar gene detection and dimensionality reduction analysis, to experimental biologists [15]. We analyzed the expression of ADHFE1 in certain cancer types (breast, colon, and gastric cancers) using the function of Boxplot in the GEPIA database.

### 2.4 Analysis of gene mutation and copy number alteration using cBioPortal

cBioPortal database is an intuitive web tool, which collects, standardizes, and delivers gene expression, somatic mutation, copy number alterations (CNAs), DNA methylation, and clinical information from 225 cancer studies in the TCGA project to the cancer researchers [16]. CNAs are generated by the GISTIC algorithm, while DNA methylation data are evaluated on the Illumina Infinium HumanMethylation450 platform. The raw data of DNA methylation are presented in the form of *β*-value, a ratio between methylated probe intensities and total probe intensities and probe-level data are normalized and condensed to a summary beta value by calculating the average methylation value for all CpG sites associated with a gene [16]. We used cBioPortal to explore whether genetic mechanisms (somatic mutation and CNVs) contribute to the altered expression of ADHFE1 in specific cancers. The genetic alteration frequency was inquired, and the expression levels of ADHFE1 with different CNV status were compared.

### 2.5 Analysis of DNA methylation using TCGA Wanderer, cBioPortal and Gene Expression Omnibus (GEO)

TCGA Wanderer (http://maplab.cat/wanderer) is an intuitive web tool allowing straightforward access and visualization of gene expression and DNA methylation profiles from TCGA [17]. Using TCGA Wanderer, we compared the methylation levels of ADHFE1 between tumor and normal tissues. Since insufficient normal samples (two cases) were evaluated for DNA methylation in patients with gastric cancer from TCGA, we searched an alternative dataset, namely GSE30601 [18,19], from GEO (https://www.ncbi.nlm.nih.gov/geo/), which is a public functional genomics data repository [20], to compare the differential methylation level of ADHFE1 between normal and gastric cancer tissues. Pearson correlation analysis to examine the relativity between ADHFE1 expression and DNA methylation in cancer samples was performed using TCGA datasets within cBioPortal.

### 2.6 Survival analysis using R2 and SurvExpress

The R2: Genomics Analysis and Visualization Platform (R2; https://r2.amc.nl/), developed by Jan Koster in the Academic Medical Center Amsterdam, is a web-based platform for genomics analysis and visualization. Patients with cancer of a selected cohort within the R2 platform were stratified by the differential expression level of ADHFE1 using the “scan” cutoff modus, and the survival analysis was carried out using the Kaplan–Meier Scanner function. SurvExpress is a web-based biomarker validation tool (http://bioinformatica.mty.itesm.mx:8080/Biomatec/SurvivaX.jsp), where the prognostic index of each patient in the selected cancer study is determined by the Cox survival analysis, and patients within the selected study cohort are classified into high-risk or low-risk subgroup according to the median prognostic index [21]. Expression levels of ADHFE1 in high- and low-risk subgroups were compared to validate its predictive effect in the survival of patients.

### 2.7 Exploring ADHFE1-relevant pathways and biological processes

Positively and negatively correlated genes to ADHFE1 were explored in TCGA datasets of the three selected cancer types (breast, colon, and gastric cancers) using the R2 platform with the Correlate Gene function. Genes with |Pearson coefficient| >0.3 and *P*-value <0.05 were collected for each cancer type. After that, the shared gene set was determined by drawing a Venn diagram. Gene enrichment analysis for common correlated gene set (positively or negatively co-expressed) was carried out using Metascape, a well-recognized and web-based platform for gene annotation (http://metascape.org) [22]. We also performed protein–protein interaction (PPI) analysis utilizing the STRING database (https://www.string-db.org) to find ADHFE1 relevant networks at the protein level. The single protein of ADHFE1 was used as the searching input, and active protein interaction sources were from textmining, experiments, databases, co-expression, neighborhood, gene fusion, and co-occurrence. The minimum required interaction score was set at 0.400 (medium confidence).

### 2.8 Statistical analysis

Bar and dot plots were drawn using GraphPad Prism version 7 (GraphPad Software, La Jolla, CA, USA). Survival curves were constructed using Kaplan–Meier Scanner within the R2 platform, and the results are displayed with *P*-values obtained from a log-rank test. The levels of significance (*P*-values) of the Oncomine, GEPIA, and SurvExpress data were determined by the programs used for the analyses. The methylation data were retrieved from TCGA Wanderer, and an unpaired *t*-test was performed to analyze two groups (normal vs cancer) using GraphPad Prism 7 software. The relativity between mRNA expression and DNA methylation of ADHFE1 in cancer samples and the NCI-60 cell lines were examined by performing Pearson correlations. The results were considered significant at *P* < 0.05.

## 3 Results

### 3.1 ADHFE1 mRNA expression and DNA methylation in cancer cell lines

**Figure 1:**
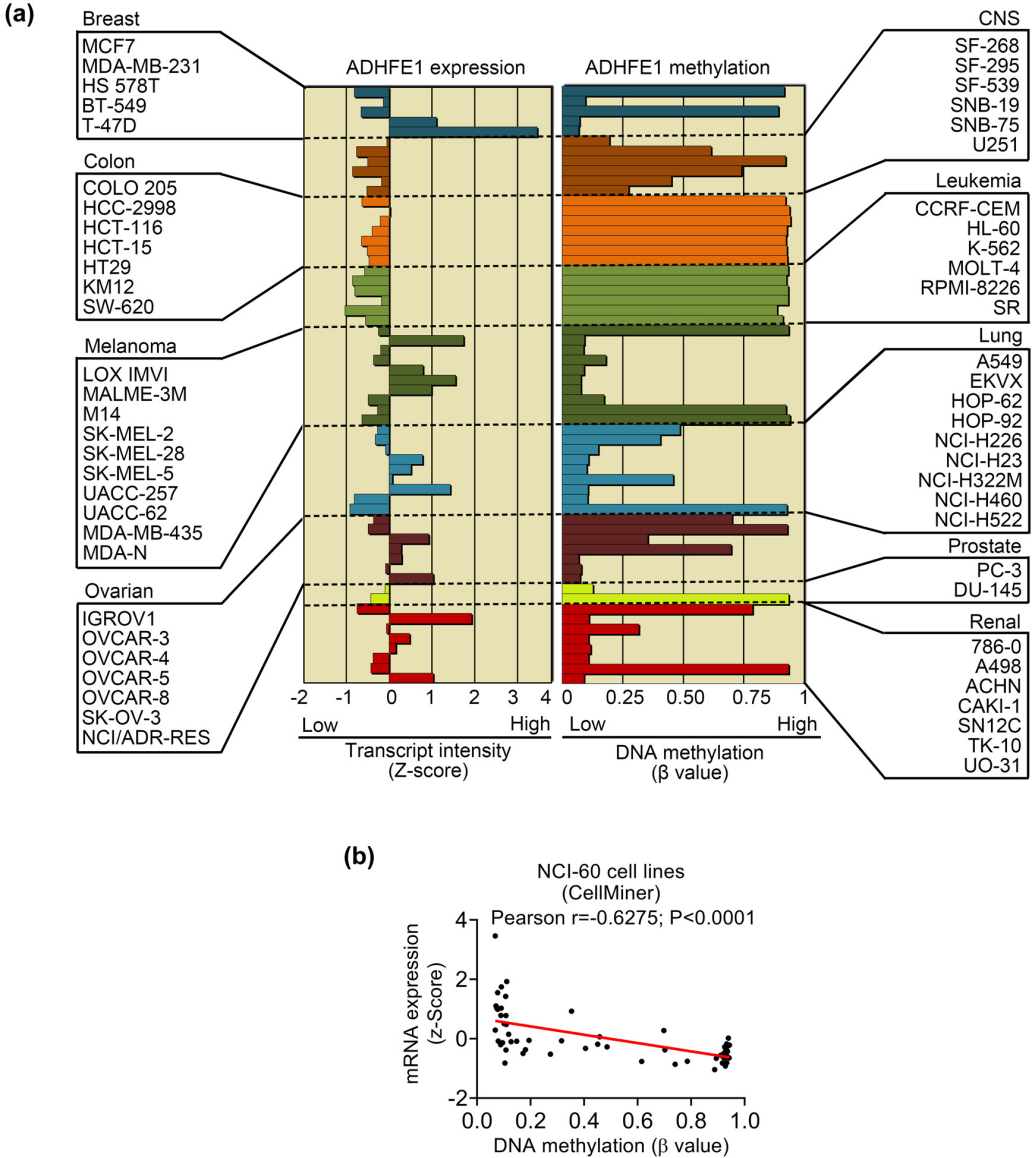
ADHFE1 expression is associated with DNA methylation in cancer cell lines. (a) Expression and methylation of ADHFE1 in NCI-60 cell line set. Data were retrieved from the CellMiner database. (b) Correlation between ADHFE1 expression and methylation in NCI-60 cell line set.

Dysregulation of ADHFE1 was observed in several types of human cancer, and DNA methylation might be a major contributor [7,8,9,23,24,25,26]. Thus, we first investigated ADHFE1 expression and DNA methylation in the NCI-60 cell line set using the CellMiner database to explore the possible role of ADHFE1 in cancer tentatively. Consistent with previous findings, downregulation and hypermethylation of ADHFE1 were found in some of the cell lines of the nine cancer types included in the CellMiner database (Figure 1a), and a negative correlation between DNA methylation and mRNA expression of ADHFE1 was observed (Figure 1b).

### 3.2 The expression pattern of ADHFE1 across cancers

**Figure 2:**
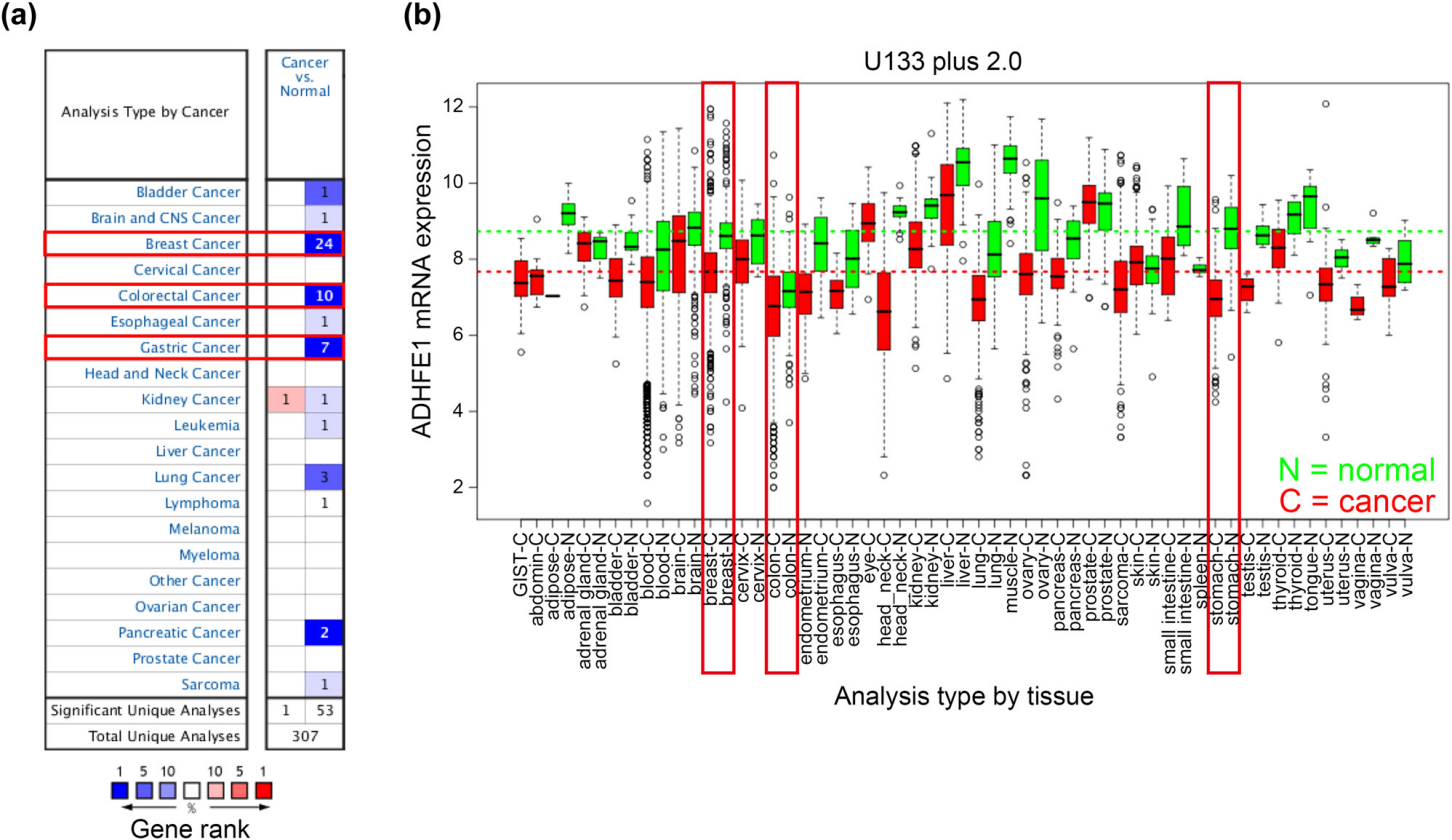
The expression pattern of ADHFE1 across cancers. (a) The expression of ADHFE1 across a range of human cancers was examined in the Oncomine database with the threshold parameters of |fold-change| >2 and *P*-value <0.05. The number of datasets with statistically significant mRNA over-expression (red) or under-expression (blue) of ADHFE1 (cancer vs corresponding normal tissue) was shown in different cancer types. Cell color is determined by the best gene rank percentile for the analyses within the cell and an analysis may be counted more than one cancer type. (b) The expression pattern of ADHFE1 mRNA in normal and tumor tissues was validated in GENT database. Boxes represent the median and the 25th and 75th percentiles, and dots represent outliers. Red boxes represent tumor tissues; green boxes represent normal tissues. Red and green dashed lines represent the average value of all tumor and normal tissues, respectively.

Next, we examined the expression pattern of ADHFE1 across a range of cancer samples using the Oncomine database. Compared to the expression level in corresponding normal tissues, ADHFE1 was downregulated in almost all types of cancer tissues examined, especially in breast, colon, and gastric cancers with relatively more significant unique analyses and higher gene rank, and only one study of kidney cancer showed upregulated expression of ADHFE1 (Figure 2a). To validate our findings in the Oncomine database, we inquired the expression of ADHFE1 in GENT, another standard online bioinformatics platform providing the expression patterns of genes across a wide range of cancer and normal tissues. In the analysis using Affymetrix Human Genome U133 Plus 2.0 Array within the GENT database, ADHFE1 expression was downregulated in nearly all cancers including breast, colon, and stomach, among others, and the average expression of ADHFE1 was lower in cancer tissues of different cancer types than that in the normal tissues (Figure 2b). The results obtained from Oncomine and GENT databases suggested that the expression of ADHFE1 is commonly downregulated in cancer tissues, and the expression pattern seems to manifest in cancers including breast, colon, and gastric cancers. Therefore, we chose the above three cancers for further study.

### 3.3 ADHFE1 expression and its correlation to patient survival in breast cancer

**Figure 3:**
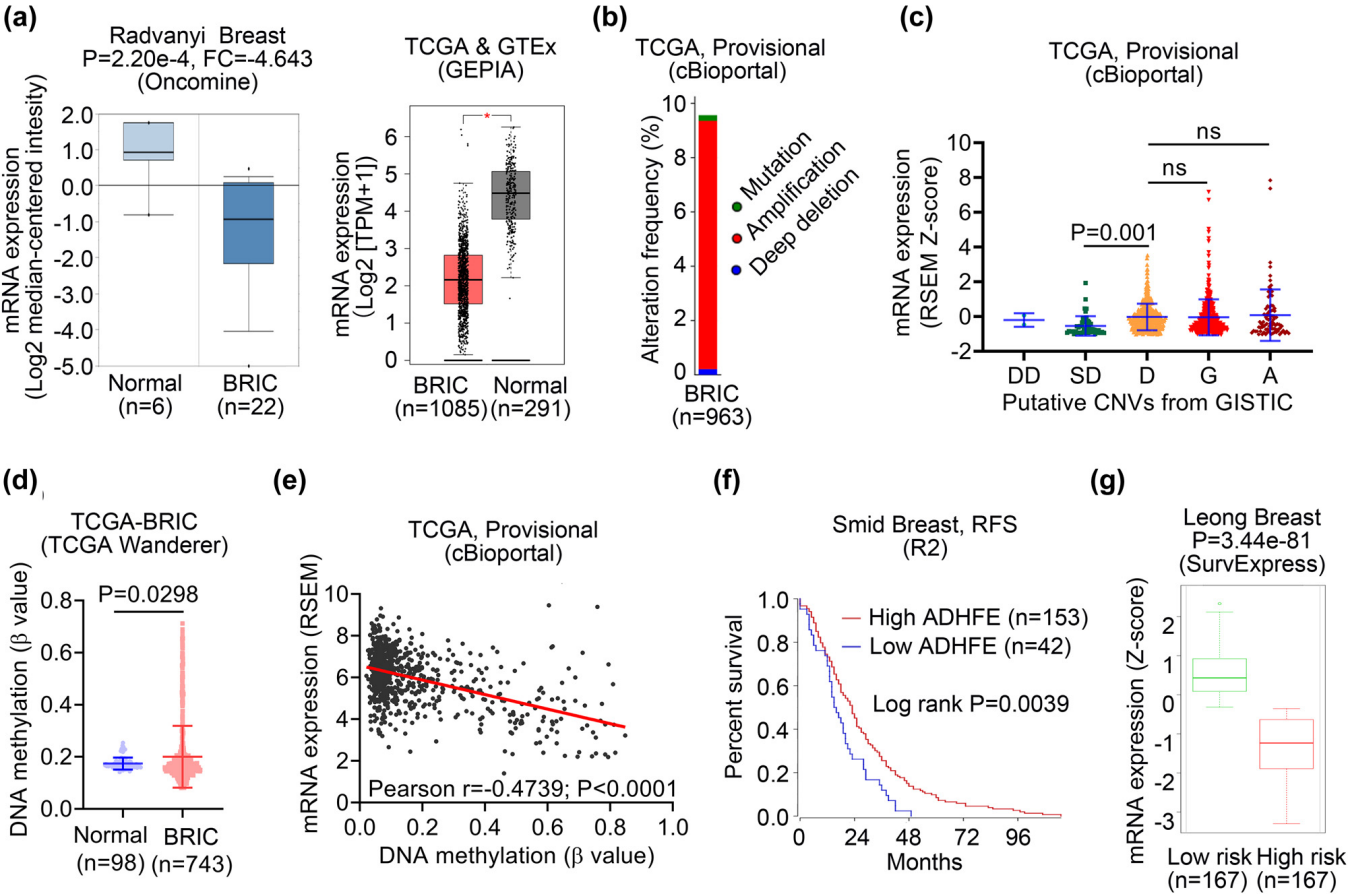
ADHFE1 expression and its correlation to patient survival in breast cancer. (a) Box-plots comparing detailed ADHFE1 expression between normal and breast cancer tissues. Expression data were retrieved from Radvanyi Breast (Oncomine database, left panel) and TCGA-BRIC (GEPIA database, right panel) datasets, respectively. *P < 0.05. (b) Genetic alterations (somatic mutation, amplification and deep deletion) of ADHFE1 gene in BRIC (TCGA, Provisional). Data were obtained from cBioPortal database. (c) The expression levels of ADHFE1 in breast cancer tissues with different status of CNVs analyzed using TCGA-BRIC dataset retrieved from cBioPortal database. (d) Box-plot showing methylation levels of normal breast (blue) and BRIC (red) tissues. Data were obtained from the TCGA Wanderer database. (e) The association between ADHFE1 mRNA expression and DNA methylation in TCGA-BRIC tissues within cBioPortal database. (f) The survival curve comparing patients with high (red) and low (blue) expression in Smid dataset was plotted from the R2 database. Difference between survival curves was analyzed using the log-rank test. (g) Box plot generated with SurvExpress biomarker validation tool showing ADHFE1 expression in low-risk (green) and high-risk (red) breast cancer patients using Leong Breast cohort. BRIC, breast invasive carcinoma; FC, fold change; DD, deep deletion; SD, shallow deletion; D, diploid; G, gain; A, amplification; ns, no significance; RFS, relapse-free survival.

We inquired detailed datasets of breast cancer studies from Oncomine and GEPIA to examine the expression of ADHFE1 in breast cancer tissues and the normal counterparts. In the Radvanyi Breast dataset, downregulation of ADHFE1 was observed in the breast cancer tissues (fold change = −4.643, *P* = 2.20 × 10^−4^; Figure 3a, left panel). Expression of ADHFE1, analyzed using the TCGA and GTEx datasets from GEPIA, was also found to be significantly downregulated in breast cancer compared to the normal breast tissues (*P* < 0.05; Figure 3a, right panel). Genetic and epigenetic alterations are recognized as two main regulatory mechanisms contributing to the abnormal expression of key genes in the initiation and development of cancer [27]. Therefore, we investigated the frequency of ADHFE1 gene mutation and CNAs in breast invasive carcinoma (BRIC; TCGA Provisional dataset, cBioPortal) to explore whether genetic mechanisms play a role in the downregulation of ADHFE1 in breast cancer. The proportions of ADHFE1 gene mutation, copy number amplification, and deep deletion were 0.21, 9.14, and 0.21%, respectively (Figure 3b). Moreover, BRIC tissues with shallow deletion showed significantly transcriptional downregulation of ADHFE1 (*P* = 0.001; Figure 3c), while gain and amplification of ADHFE1 copy number had no influence on gene expression (both *P* > 0.05, Figure 3c). DNA methylation is the most extensively studied epigenetic modification, acting as the key element and is classically responsible for transcriptional silence via hypermethylation of CpG islands located in the promoter region of a certain gene [28]. Analysis of the methylation data from the TCGA-BRIC dataset deposited by the TCGA Wanderer database showed a higher level of methylation quantified by methylation *β* value in breast cancer tissues compared to that in normal breast tissues (*P* = 0.0298; Figure 3d). Furthermore, we observed a negative association between ADHFE1 mRNA expression and DNA methylation in BRIC tissues (Pearson *r* = −0.4739, *P* < 0.0001; Figure 3e). These results suggested that DNA methylation may contribute to ADHFE1 downregulation in breast cancer while CNV may not be a major contributor considering its low frequency. In addition, we compared the survival of patients stratified by ADHFE1 expression using the R2 platform. In the Smid dataset, patients with low expression of ADHFE1 had significantly shorter relapse-free survival compared to those with high expression of ADHFE1 (log-rank *P* = 0.0039; Figure 3f). We also validate the prognostic value of ADHFE1 using SurvExpress, an online biomarker validation tool. Patients with breast cancer of the entire Leong dataset summarized in the SurvExpress database were classified into low-risk and high-risk subgroups according to the median prognostic index determined by Cox regression analysis, and the low-risk subgroup tended to have a higher expression of ADHFE1 than the high-risk subgroup (*P* = 3.44 × 10^−81^; Figure 3g). Collectively, above data-driven results suggest that DNA methylation are associated with the downregulation of ADHFE1 in breast cancer, and decreased ADHFE1 is a risky factor of patient survival in breast cancer.

### 3.4 ADHFE1 expression and its correlation to patient survival in colon cancer

**Figure 4:**
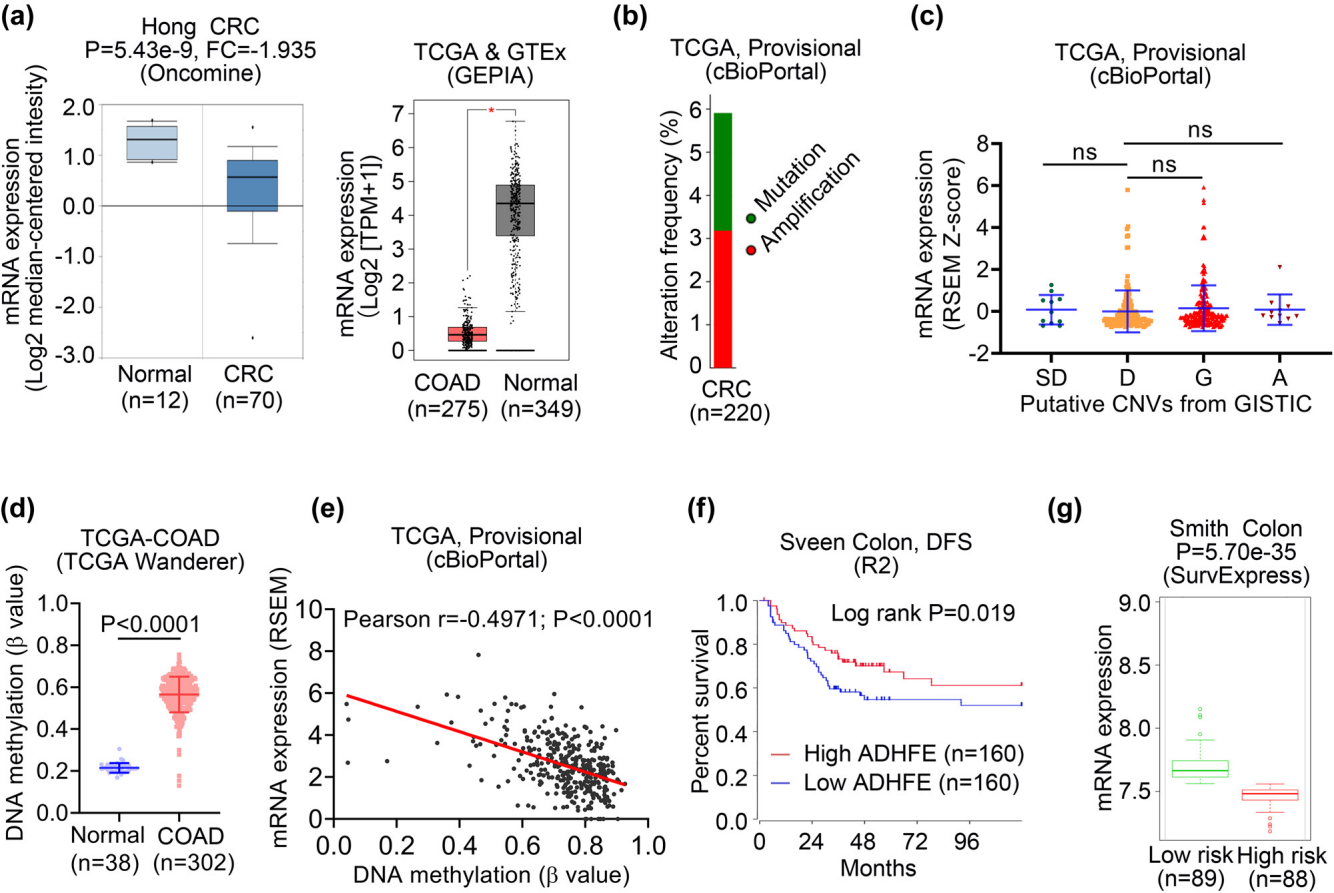
ADHFE1 expression and its correlation to patient survival in colon cancer. (a) Box plots comparing detailed ADHFE1 expression between normal and colon cancer tissues. Expression data were retrieved from Hong CRC (Oncomine database, left panel) and TCGA-COAD (GEPIA database, right panel) datasets, respectively. *P < 0.05. (b) Genetic alterations (somatic mutation, amplification, and deep deletion) of ADHFE1 gene in CRC (TCGA, Provisional). Data were obtained from cBioPortal database. (c) The expression levels of ADHFE1 in CRC tissues with different status of CNVs analyzed using TCGA-CRC dataset retrieved from cBioPortal database. (d) Box plot showing methylation levels of normal breast (blue) and COAD (red) tissues. Data were obtained from the TCGA Wanderer database. (e) The association between ADHFE1 mRNA expression and DNA methylation in TCGA-CRC tissues within cBioPortal database. (f) The survival curve comparing patients with high (red) and low (blue) expression in Sveen Colon dataset was plotted from the R2 database. Difference between survival curves was analyzed using the log-rank test. (g) Box-plot generated with SurvExpress showing ADHFE1 expression in low-risk (green) and high-risk (red) patients with colon cancer using Smith Colon cohort. CRC, colorectal cancer; FC, fold change; COAD, colon adenocarcinoma; SD, shallow deletion; D, diploid; G, gain; A, amplification; ns, no significance; DFS, disease-free survival.

CRC ranks third in cancer incidence and second in cancer-related death worldwide [1], with diverse underlying molecular features and, thus, heterogeneous clinical outcomes. Dysregulation and hypermethylation of ADHFE1 were reported in CRC patient tissues and cell lines [8,9,29,30,31]. However, the correlation between ADHFE1 expression and patient survival in colon cancer has not been investigated. Significant downregulation of ADHFE1 in patients with colon cancer was observed in the Hong CRC (Oncomine), TCGA-Colon Adenocarcinoma (COAD), and GTEx (GEPIA) datasets (both *P* < 0.05; Figure 4a). We then investigated whether genetic alterations contributed to the downregulation of ADHFE1 expression in colon cancer. Analysis of the TCGA-CRC dataset (TCGA Provisional, cBioPortal) revealed a low mutation (2.73%) and CNA (amplification, 3.18%; deep deletion, 0.00%) frequency in CRC (Figure 4b), and no association was found between CNA status of ADHFE1 and transcriptional expression (all *P* > 0.05, Figure 4c). On the other hand, an increased methylation level of ADHFE1 was found in colon cancer tissues compared to their normal counterparts (TCGA-COAD, TAGA Wanderer; *P* < 0.0001; Figure 4d). We also observed a negative correlation between ADHFE1 methylation and gene expression (TCGA-CRC, Provisional, cBioPortal; Pearson *r* = −0.4971, *P* < 0.0001; Figure 4e). Survival analysis with the Sveen dataset showed that the low expression group had significantly poorer disease-free survival than the high expression group (R2; log-rank *P* = 0.019; Figure 4f). In the Smith dataset, ADHFE1 expression of patients in the high-risk subgroup was significantly lower than those in the low-risk subgroup (*P* = 5.70 × 10^−35^; Figure 4g). These data suggested that the inactivation of ADHFE1 in colon cancer is associated with DNA methylation and correlates with elevated cancer risk.

### 3.5 ADHFE1 expression and its correlation to patient survival in gastric cancer

**Figure 5:**
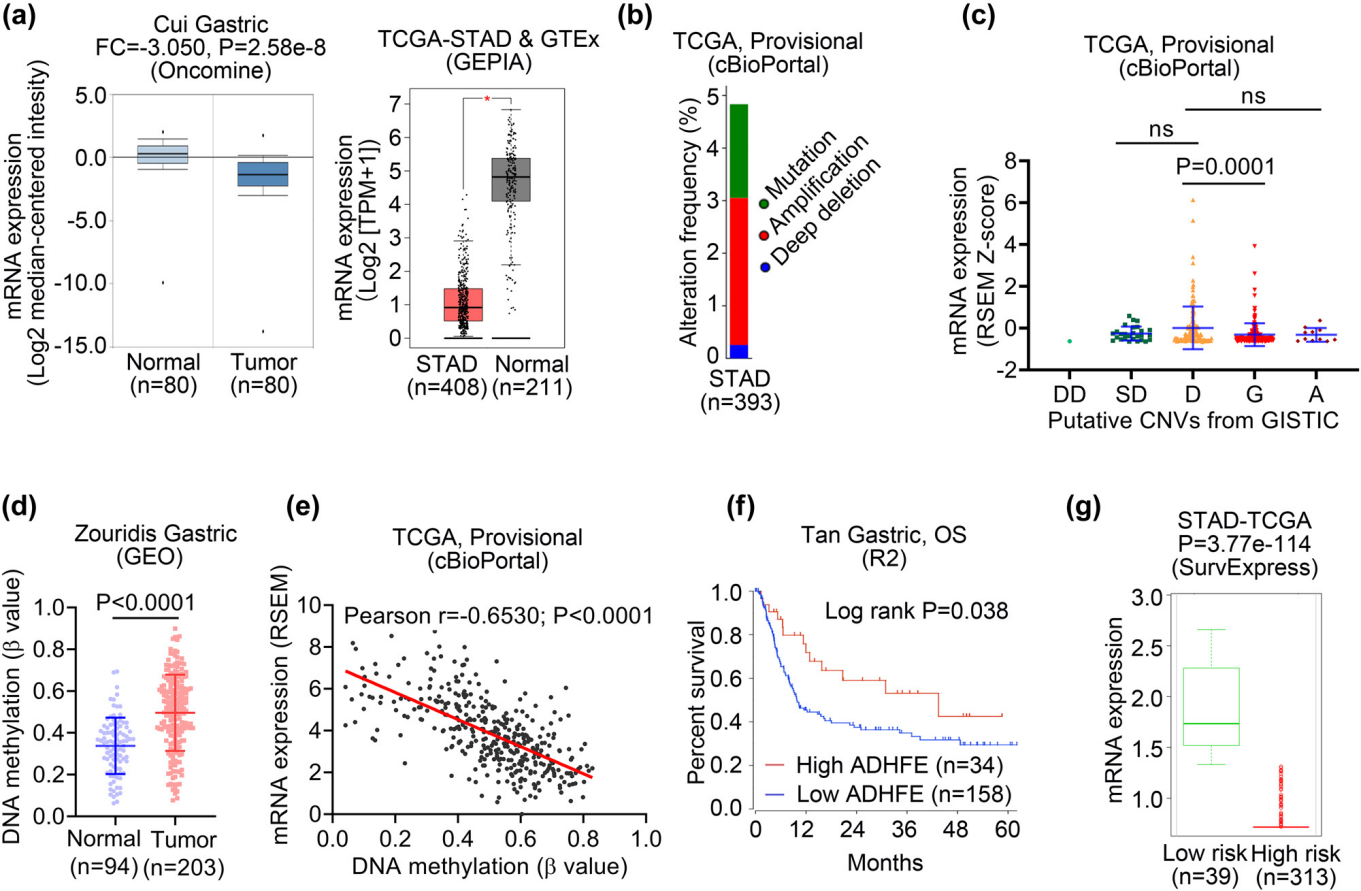
ADHFE1 expression and its correlation to patient survival in gastric cancer. (a) Box plots comparing detailed ADHFE1 expression between normal and gastric cancer tissues. Expression data were retrieved from Cui Gastric (Oncomine database, left panel) and TCGA-STAD (GEPIA database, right panel) datasets, respectively. *P < 0.05. (b) Genetic alterations (somatic mutation, amplification, and deep deletion) of ADHFE1 gene in gastric cancer (TCGA-STAD, Provisional). Data were obtained from cBioPortal database. (c) The expression levels of ADHFE1 in gastric cancer tissues with different status of CNVs analyzed using TCGA-STAD dataset retrieved from cBioPortal database. (d) Box plot showing methylation levels of normal gastric (blue) and gastric cancer (red) tissues. Data were obtained from Zouridis Gastric dataset within the GEO database. (e) The association between ADHFE1 mRNA expression and DNA methylation in gastric cancer tissues within cBioPortal database. (f) The survival curve comparing patients with high (red) and low (blue) expression in Tan Gastric dataset was plotted from the R2 database. Difference between survival curves was analyzed using the log-rank test. (g) Box plot generated with SurvExpress showing ADHFE1 expression in low-risk (green) and high-risk (red) patients with gastric cancer using TCGA-STAD cohort. STAD, stomach adenocarcinoma; FC, fold change; DD, deep deletion; SD, shallow deletion; D, diploid; G, gain; A, amplification; ns, no significance; OS, overall survival.

ADHFE1 mRNA expression was downregulated in gastric cancer tissues compared to the normal counterparts according to both Oncomine and GENT databases (Figure 2). From the detailed analysis, decreased expression of ADHFE1 was found in Cui Gastric (Oncomine) and TCGA-Stomach Adenocarcinoma (STAD) and GTEx (GEPIA) datasets (both *P* < 0.05; Figure 5a). We checked the alteration frequency of mutation and CNV in the TCGA-STAD dataset (TCGA Provisional, cBioPortal). The total alteration frequency was 4.83% and deep deletion accounted for only 0.25% (Figure 5b). Moreover, no association was found between the expression level of ADHFE1 and CNV status from diploid to shallow deletion (TCGA Provisional, cBioPortal; *P* > 0.05; Figure 5c). Intriguingly, the expression of ADHFE1 in gastric cancer tissues with a gain of copy number was significantly lower than those with diploid ADHFE1 (*P* = 0.0001; Figure 5c), suggesting alternative regulatory mechanisms potently contribute to transcriptional downregulation of ADHFE1 in gastric cancer. Analysis of the methylation data of the Zouridis dataset retrieved from the GEO database showed significantly higher level of methylation in gastric cancer tissues when compared with normal gastric tissues (*P* < 0.0001; Figure 5d). Furthermore, mRNA expression of ADHFE1 was negatively associated with and DNA methylation in gastric cancer tissues (TCGA Provisional, cBioPortal; Pearson *r* = −0.6530, *P* < 0.0001; Figure 5e). Survival analysis of the Tan dataset using the R2 platform showed significantly shorter patient survival in the low ADHFE1 expression group when compared with the high ADHFE1 expression group (log-rank *P* = 0.038; Figure 5f). We validated the prognostic value of ADHFE1 in the TCGA-STAD dataset using SurvExpress that patients with gastric cancer in low-risk subgroup had higher expression of ADHFE1 than those in the high-risk subgroup (*P* = 3.77 × 10^−114^; Figure 5g). These results suggest that gastric cancer has significant ADHFE1 downregulation which is significantly related to DNA methylation but not CNA, and ADHFE1 expression is negatively correlated with the overall survival of patients with gastric cancer.

### 3.6 Exploring ADHFE1-relevant pathways and biological processes

**Figure 6:**
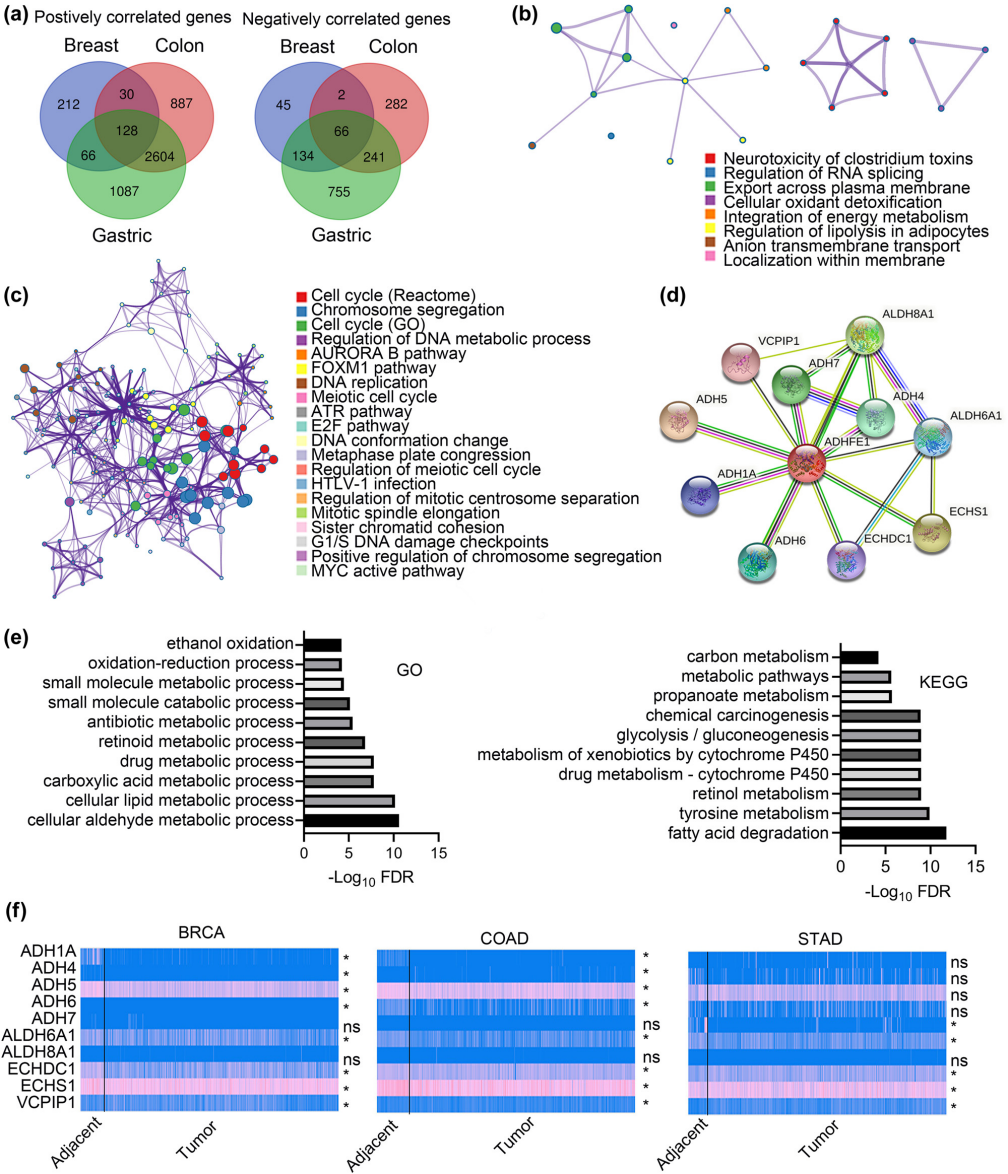
Exploring ADHFE1-relevant pathways and biological processes. (a) Venn diagrams of genes positively and negatively correlated to ADHFE1, showing commonly shared gene sets in breast, colon and gastric cancers. (b) & (c) Gene enrichment analysis of the positively (b) and negatively (c) correlated gene sets shared by above three cancer types using Metascape. Each node represents one enriched term. Node size is proportional to the total number of genes within each term. Proportion of shared genes between gene sets is represented as the thickness of the line between nodes. (d) PPI analysis of ADHFE1 relevant network at the protein level using STRING database. (e) GO and KEGG enrichment analysis of the signaling pathways of the protein members in ADHFE1 relevant network. (f) The expression levels of the coding genes of the protein members in ADHFE1 relevant network in the TCGA cohort of BRCA, COAD, and STAD. PPI, protein-protein interaction; GO, Gene Ontology; KEGG, Kyoto Encyclopedia of Genes and Genomes; FDR, false discovery rate; BRCA, breast cancer; COAD, colon adenocarcinoma; STAD, stomach adenocarcinoma.

Finally, we set to explore potential signaling pathways and biological processes related to dysregulated ADHFE1 expression in cancer. We analyzed transcriptome data of the above three types of cancers, namely, breast, colon, and gastric cancers, from TCGA datasets through the R2 platform. We used the Correlate Gene function to find genes significantly correlated with ADHFE1 expression in each cancer type (|Pearson coefficient| >0.3 and *P*-value <0.05). The identified positively and negatively correlated gene sets that were commonly shared by the three types of cancers contained 128 and 66 individual genes, respectively (Figure 6a). Then, the two gene sets identified were subjected to gene enrichment analysis using Metascape. The results showed that the positively correlated genes were mainly categorized in pathways related to cellular detoxification and energy metabolism (Figure 6b), and these functions of ADHFE1 were reported by previous studies [6,32]. The negatively correlated genes were mainly enriched in biological processes related to DNA replication and cell cycle regulation (Figure 6c). We also performed PPI analysis utilizing the STRING database to find ADHFE1 relevant network at the protein level. A network consisting of 11 proteins (ADHFE1 included) was revealed (Figure 6d), and the pathway analysis of these proteins showed that they were mainly involved in the processes of oxidation and metabolism, which were consistent with the enriched terms by ADHFE1 correlated genes at the transcriptional level (Figure 6e). Furthermore, we profiled the expression of the coding genes of these proteins in the TCGA cohort of BRCA, COAD, and STAD, and the results showed that these genes were more or less abnormally expressed in the above cancers (Figure 6f). These findings suggested that dysregulated ADHFE1 (or ADHFE1 relevant networks) may be associated with certain key pathways related to energy metabolism, DNA replication, and cell cycle regulation in cancer progression.

## 4 Discussion

ADHFE1 is a member of the iron-activated alcohol dehydrogenase family that plays multiple roles in various physiological processes [6,27,32,33]. Several studies have also shown that ADHFE1 was involved in cancer development [7,8,9,10,24,34]. However, the functional role of ADHFE1 and its impact on cancer prognosis are not fully understood, and some studies reported controversial results regarding the role of ADHFE1 in different types of cancers. For example, it has been reported that ADHFE1 is downregulated and hypermethylated in CRC tissues, and high ADHFE1 is significantly associated with good differentiation of CRC [8]. However, ADHFE1 was identified as an MYC-linked oncogene that induces metabolic reprogramming and cellular de-differentiation in breast cancer [10]. We proposed that the contradictory role of ADHFE1 in differing cancer types might be attributed to its multiple roles in cellular functionalities (such as metabolic reprogramming, DNA replication, and cell cycle control), which depend on the cancer type and cellular status.

A variety of genetic alterations and epigenetic changes play an important role in cancer initiation and progression. Multiple genomic platforms can broadly survey gene expression and DNA methylation, as evidenced by TCGA project, which may aid us in exploring novel biomarkers in cancer. Therefore, in the present study, we have systematically analyzed ADHFE1 expression and the underlying regulatory mechanisms in various cancers through several recognized expression databases and bioinformatics tools. We first explored ADHFE1 mRNA expression and DNA methylation of ADHFE1 in the NCI-60 cell line set using the CellMiner database. Next, we performed the analysis with cancer tissue samples in the Oncomine and GENT databases and revealed that expression of ADHFE1 is commonly downregulated in cancer tissues compared with normal tissues, suggesting ADHFE1 as a promising diagnostic biomarker in cancer. Since the expression pattern of ADHFE1 seems to manifest in cancers including breast, colon, and gastric cancers, among others, we chose these three cancer types for subsequent analysis. Assessing the methylation data from cBioPortal and TCGA Wanderer platforms, we found that ADHFE1 was commonly hypermethylated in these three types of cancers, and methylation level of ADHFE1 was negatively correlated with ADHFE1 mRNA expression, suggesting that DNA methylation may be a major contributor to ADHFE1 inactivation in cancer. Next, we investigated the association between the expression level of ADHFE1 and patient survival in various cancers using R2 and SurvExpress. In general, low ADHFE1 expression was associated with poor survival. Finally, we identified genes correlated with ADHFE1 shared by the three selected cancers, based on which gene enrichment analysis was performed to explore ADHFE1 affected pathways. Dysregulation of ADHFE1 is involved in pathways, including energy metabolism, DNA replication, and cell cycle regulation, among others, suggesting its potential role in active biological processes related to cancer progression. Moreover, the PPI analysis revealed a consistent pathway enrichment result at the protein level. Collectively, the above results suggested that ADHFE1 is frequently silenced by DNA methylation in human cancers and may also act as a promising biomarker predictive of patient survival.

## 5 Conclusion

**Figure 7:**
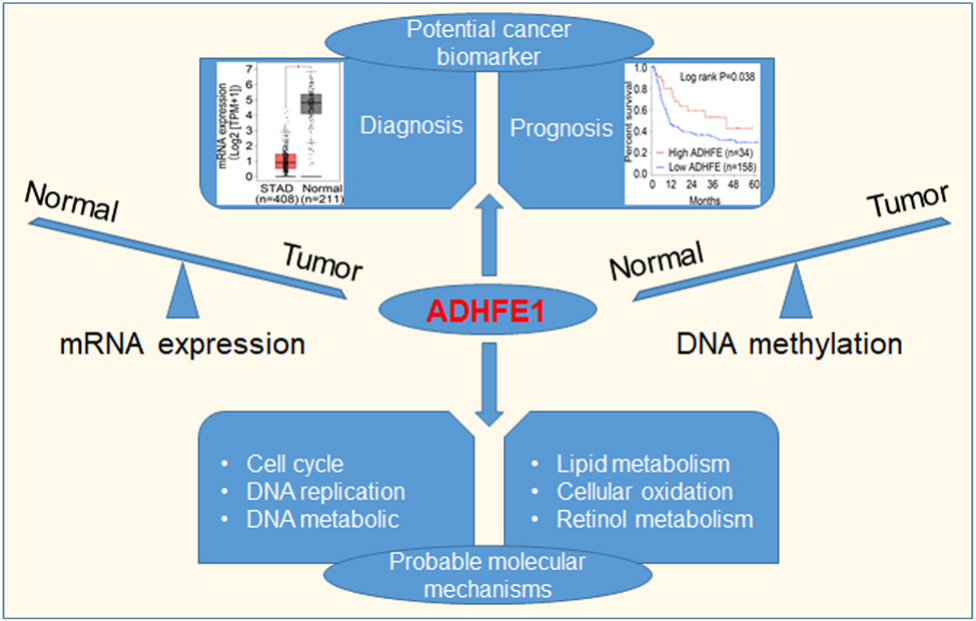
Summary of the study. ADHFE1 expression is regulated by DNA methylation which can be a promising diagnostic and prognostic biomarker in cancer; dysregulated ADHFE1 may participate in cancer progression by regulation of metabolic process, DNA replication, and cell cycle, among others.

In summary, our findings demonstrated that ADHFE1 expression is regulated by DNA methylation and can be a promising diagnostic and prognostic biomarker in cancer. Moreover, dysregulated ADHFE1 might participate in cancer progression through involvement in signaling pathways, including energy metabolism, DNA replication, and cell cycle regulation, among others; nevertheless, experimental and clinical studies are greatly needed to clarify the detailed molecular mechanisms and elaborate its potential utility (Figure 7).
